# Noninvasive direct detection of ocular mucositis by in vivo confocal microscopy in patients treated with S-1

**Published:** 2009-12-26

**Authors:** Tai-ichiro Chikama, Norihisa Takahashi, Makiko Wakuta, Teruo Nishida

**Affiliations:** Department of Ophthalmology, Yamaguchi University Graduate School of Medicine, Ube, Yamaguchi, Japan

## Abstract

**Purpose:**

S-1 is an oral antineoplastic agent that contains a prodrug of 5-fluorouracil and has adverse effects on skin, alimentary tract mucosa, and the ocular surface. We investigated the effects of S-1 on the corneal epithelium by in vivo confocal microscopy and histopathologic analysis.

**Methods:**

Twelve patients with eye problems related to S-1 treatment participated in the study. Twenty eyes of ten subjects were evaluated by laser-scanning confocal microscopy. Corneal epithelial debridement as a diagnostic therapy and histopathologic analysis were performed for five eyes of three subjects affected in the pupillary zone of the cornea.

**Results:**

Slitlamp examination revealed a local limbal abnormality characterized by epithelial invasion toward the center of the cornea in all 24 eyes. In vivo confocal microscopy revealed an altered structure of the corneal epithelium with abnormal epithelial cells and inflammation. One of five specimens subjected to cytologic diagnosis showed moderate dysplasia. Hematoxylin and eosin staining showed that each abnormal epithelial sheet lacked the stratified structure of the normal corneal epithelium. Immunofluorescence analysis revealed the presence of cells positive for one, both, or neither of cytokeratins 12 and 4 in each lesion.

**Conclusions:**

S-1 can induce ocular mucositis with dysplasia, likely affecting cellular functions, including differentiation, of the corneal epithelium. In vivo confocal microscopy allowed the noninvasive detection of cellular changes in the cornea as an adverse effect of S-1 administration.

## Introduction

Oral and gastrointestinal mucositis is a frequent adverse effect of chemotherapy or radiotherapy for cancer. 5-Fluorouracil (5-FU) is a common chemotherapeutic agent, the administration of which is associated with severe mucositis [1]. S-1 is an oral antineoplastic drug that is an active combination of tegafur (a prodrug of 5-FU), gimeracil, and oteracil [2]. It is more active and less toxic than intravenous 5-FU, being associated with fewer gastrointestinal adverse effects. It is possible to achieve a high concentration of 5-FU in patients for a longer time with S-1, although adverse effects are still induced in sensitive organs and tissues [2,3]. Myelosuppression, skin rash, anorexia, nausea, vomiting, and fatigue have been reported as major adverse effects of S-1 therapy [2,4], with the alimentary tract mucosa and skin, both of which are multilayered epithelia consisting of cells with high metabolic and turnover rates, being especially sensitive to drug toxicity [4,5].

**Figure 1:**
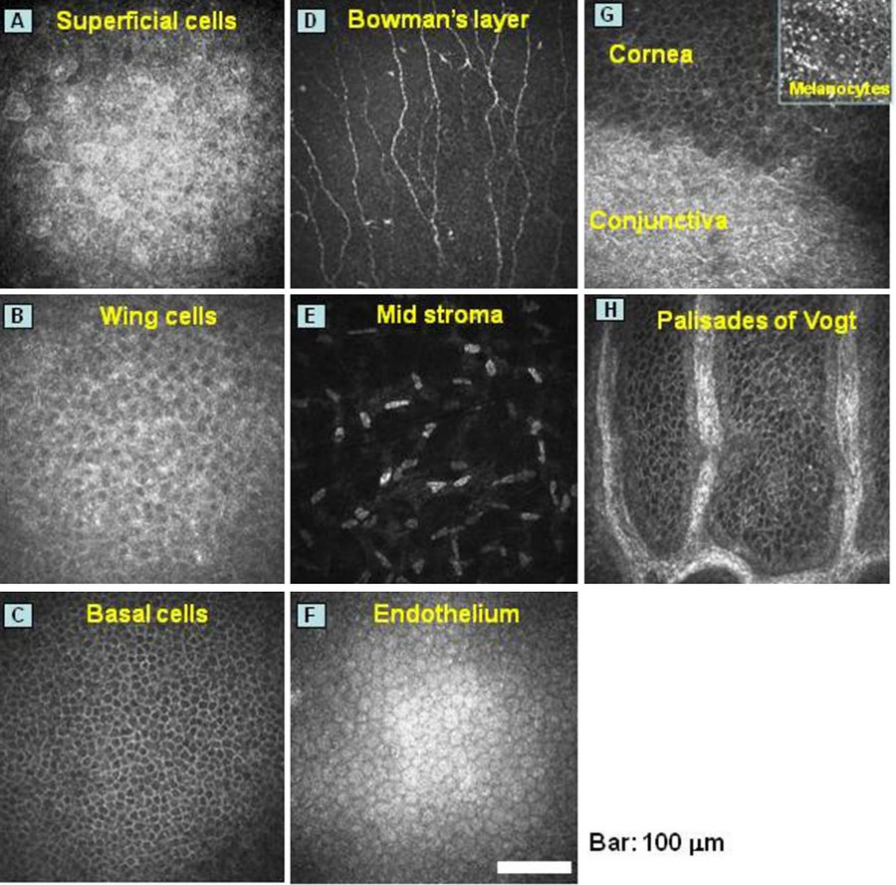
In vivo confocal microscopy of the cornea and the limbus in a normal eye with the an in vivo laser confocal microscope with an attachment for the cornea (Heidelberg Retina Tomograph II–Rostock Cornea Module, or HRTII-RCM®; Heidelberg Engineering, Heidelberg, Germany). Panels **A** through **F** represent areas of 400×400 µm corresponding to the superficial epithelial cell layer, wing cell layer, basal cell layer, Bowman’s layer, mid stroma, and endothelium, respectively. The approximate levels of the sections are indicated in the hematoxylin and eosin-stained histologic specimen. Panels **G** and **H** show the limbus at the border between the cornea and conjunctiva and the limbal palisades of Vogt, respectively. The peripheral cornea often retains melanocytes in the basal epithelial layer (inset in **G**).

The ocular surface, consisting of the cornea, conjunctiva, lacrimal system, and eyelid, is also lined with a multilayered mucosal epithelium that protects the underlying connective tissue (Figure 1). 5-FU can be detected in tears after its intravenous administration [6,7] and 5-FU in tear fluid is thought to induce ocular surface and lacrimal complications. Both tearing and blurred vision have been reported with a prevalence of >10% in patients treated with 5-FU [8]. Tearing, conjunctivitis, keratitis, eye pain, and visual disturbance are listed as ophthalmic adverse effects of S-1 administration on the package insert in Japan. As far as we are aware, however, the incidence and pathology of ocular surface complications in patients treated with S-1 have not been fully characterized, with a histopathologic investigation of S-1–induced corneal disorders not having been performed.

The development of an in vivo laser confocal microscope with an attachment for the cornea (Heidelberg Retina Tomograph II–Rostock Cornea Module, or HRTII-RCM^®^; Heidelberg Engineering, Heidelberg, Germany) has yielded new insights into ocular surface disorders by providing high-resolution images of cells at the ocular surface (Figure 1). In addition to ocular surface cells [9-12], the development of confocal optics has allowed the oral mucosa, tongue, and teeth to be examined layer by layer at the cellular level [11,13].

The purpose of the present study was to characterize specific pathologic features of the cornea in patients with S-1-induced corneal disorders with the use of in vivo laser confocal microscopy in order to provide an insight into disease pathogenesis.

## Methods

### Subjects

We undertook an evaluation of 12 patients with corneal disorders associated with S-1 (TS-1^®^; Taiho Pharmaceutical, Tokyo, Japan) administration at Yamaguchi University Hospital, Ube, Japan. All patients were referred to our hospital by their local ophthalmologists for diagnosis or treatment of their corneal epithelial disorders. All patients received S-1 as adjuvant chemotherapy. Sex, age, visual acuity, ophthalmic symptoms, and general symptoms of the patients as well as their corneal findings by slitlamp biomicroscopy and in vivo confocal microscopy were recorded. The study was approved by the Institutional Review Board of Yamaguchi University Hospital, and all patients provided informed consent to participate in the study. The protocol adhered to the principles of the Declaration of Helsinki.

### In vivo laser confocal microscopy

In vivo confocal microscopy was performed with an HRTII-RCM system (Heidelberg Engineering) as previously described [14]. In summary, a drop of local anesthetic (0.4% benoxinate hydrochloride; Santen, Osaka, Japan) was instilled into the lower fornix of each eye, and a drop of polymer gel (Viscotears; CIBA Vision, Atlanta, GA) was applied to the microscope probe to allow for optical coupling of the microscope objective lens to the cornea. Corneal lesions were scanned by alternating vertical and horizontal movement from the center to the limbus. Several confocal microscopic images of tangential optical sections of the superficial, wing, and basal cell layers of the corneal epithelium, stroma, and endothelium were obtained for each eye. Oblique sections were also obtained when abnormal findings are observed and to confirm their orientation. No complications of examination with the HRTII-RCM system were noted.

### Histopathologic analysis

Corneal epithelial debridement was performed as diagnostic therapy for the five eyes of three subjects that were affected in the pupillary zone of the cornea. Frozen sections of the lesion specimens were examined by Hematoxylin and Eosin (HE; Merck, Darmstadt, Germany) staining and immunofluorescence staining for cytokeratins. For double immunofluorescence staining of cytokeratin 12 (K12, which is specific for corneal epithelial cells) and cytokeratin 4 (K4, which shows a high specificity for conjunctival epithelial cells), sections were incubated consecutively with rabbit polyclonal antibodies to mouse K12 (University of Cincinnati, Cincinnati, OH and Wakayama Medical University, Wakayama, Japan), Alexa Fluor 488–conjugated antibodies to rabbit immunoglobulin G (Molecular Probes, Eugene, OR), mouse monoclonal antibodies to K4 (Sigma, St. Louis, MO), and Alexa Fluor 555–conjugated antibodies to mouse immunoglobulin G (Molecular Probes). Nuclei were stained with 4′,6-diamidino-2-phenylindole (Molecular Probes, Eugene, OR).

## Results

### Clinical and slitlamp biomicroscopy findings

The clinical characteristics of the 12 patients with bilateral corneal disorders are shown in Table 1. The patients included seven men and five women, with an overall mean±standard deviation (SD) age of 64.3±7.6 years (range, 52–78 years). No history of ocular surface disorders was revealed for any of the patients in individual interviews. All patients reported some type of ocular discomfort. Subjective ophthalmic symptoms included visual disturbance, tearing, and foreign body sensation in six, eight, and eight patients, respectively. Eight patients had skin problems, including increased pigmentation in seven individuals, and nail abnormalities were apparent in four patients. Subjective ophthalmic symptoms were first noticed from 1.5 to 36 months after the onset of S-1 administration, with an overall mean±SD of 7.9±10.2 months.

**Table 1:** Clinical characteristics of patients with S-1–induced ocular mucositis.

**Patient**	**Sex**	**Age (years)**	**Primary disease**	**S-1 dose (mg/day)/ treatment duration (months)/ onset of ocular symptoms (months)**	**Ocular symptoms**	**General symptoms**	**Visual acuity at first visit**	**Withdrawal of S-1 administration**	**Progress**
1	F	52	Gastric cancer	110/24/12	Bil. visual disturbance Bil. foreign body sensation	Skin pigmentation	OD = 0.05 (0.7) OS = 0.07 (1.0)	Yes	Improved OD = 0.1 (1.2) OS = 0.08 (1.5)
2	M	73	Gastric cancer	Unclear/24/2	Bil. visual disturbance	Skin pigmentation	OD = 0.2 (0.2) OS = 0.09 (0.15)	Yes	Residual epithelial disorder OD = 0.4 (0.7) OS = 0.09 (0.4)
3	M	68	Rectal cancer	100/21/2	Visual disturbance OD Bil. tearing	None	OD = 0.04 (0.05) OS = 0.6 (0.7)	Yes	Improved but duct obstruction irreversible OD = 1.0 (NC) OS = 0.7 (0.9)
4	F	58	Breast cancer	120/15/1.5	Bil. foreign body sensation	Skin dryness Nail abnormality	OD = 0.7 (1.0) OS = 0.8 (1.2)	Yes	Residual epithelial disorder OD = 0.6 (0.7) OS = 0.2 (0.5)
5	M	57	Gastric cancer	125/68/36	Bil. foreign body sensation Bil. tearing	Skin pigmentation	OD = 0.9 (1.2) OS = 0.9 (NC)	Yes	Improved but duct obstruction irreversible OD = 0.8 (0.9) OS = 1.0 (NC)
6	M	66	Gastric cancer	120/>72/4	Bil. foreign body sensation Bil. tearing	Skin pigmentation	OD = 0.8 (NC) OS = 0.8 (NC)	No	Improved slightly but duct obstruction irreversible OD = 0.7 (1.2) OS = 1.2 (NC)
7	F	72	Gastric cancer	80/9/3	Bil. visual disturbance Bil. tearing	None	OD = 0.8 (0.9) OS = 0.4 (0.4)	Yes	Improved OD = 0.8 (1.0) OS = 0.9 (NC)
8	M	60	Biliary tract cancer	100/8/6	Bil. visual disturbance Bil. tearing	Skin pigmentation Skin dryness	OD = 0.3 (0.5) OS = 0.2 (0.3)	Yes	Improved but duct obstruction irreversible OD = 0.8 (1.2) OS = 0.5 (1.2)
9	F	59	Gastric cancer	50/6/6	Bil. foreign body sensation Bil. tearing	None	OD = 0.05 (1.0) OS = 0.06 (1.0)	Yes	Improved OD = 0.05 (1.0) OS = 0.06 (1.0)
10	F	67	Colon cancer	100/12/12	Bil. foreign body sensation Bil. tearing	Nail abnormality	OD = 0.3 (0.4) OS = 0.4 (0.6)	Yes	Improved OD = 0.9 (NC) OS = 1.0 (NC)
11	M	62	Gastric cancer	125/3/2	Bil. foreign body sensation Bil. tearing	Skin dryness Skin pigmentation Nail abnormality	OD = 0.7 (0.7) OS = 0.15 (0.2)	Yes	Improved OD = 0.5 (0.8) OS = 0.3 (0.5)
12	M	78	Lung cancer	100/3/2	Bil. visual disturbance Bil. foreign body sensation	Skin pigmentation Nail abnormality	OD = 0.3 (0.7) OS = 0.4 (NC)	Yes	Improved OD = 0.5 (0.7) OS = 0.9 (NC)

In the table, "Bil." indicates bilateral. Visual acuity values in parentheses are corrected; NC, non corrigible. Progress refers to symptoms of ocular mucositis at various times after discontinuation of S-1 treatment or at time of last follow-up (patient 6); visual acuity values are also given for these times.

**Figure 2:**
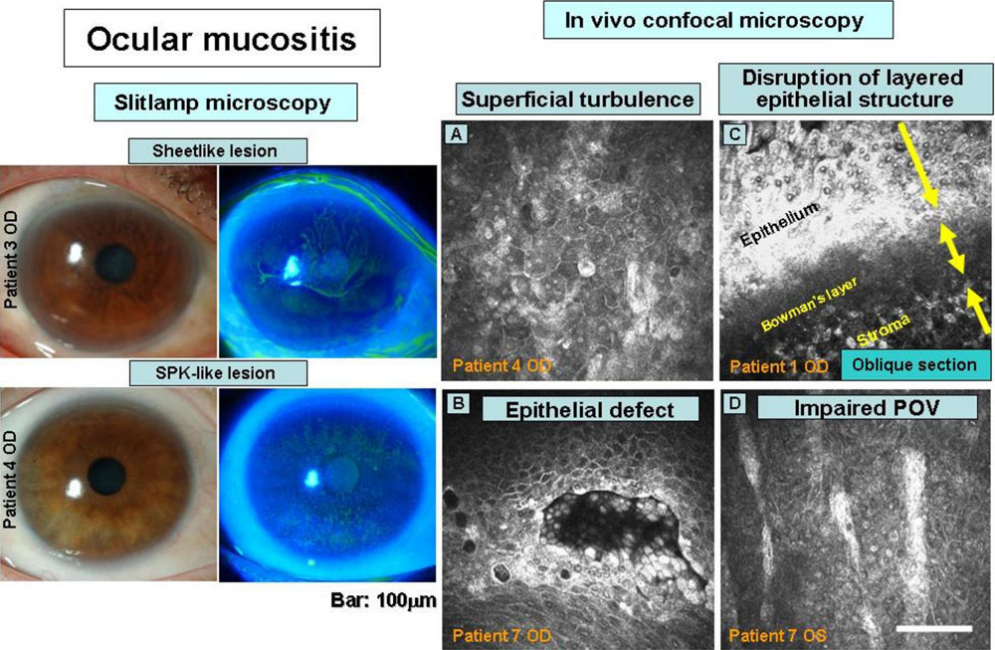
Ocular mucositis revealed by slitlamp and in vivo confocal microscopy. Slitlamp microscopy (left panels) revealed corneal epithelial abnormalities characterized by two patterns of turbulence (sheet-like or superficial punctate keratopathy-like (SPK-like) lesions) that were highlighted by fluorescein staining. Confocal microscopy (right panels) revealed nonuniformity in size and intensity of superficial epithelial cells (**A**), cellular defects in the superficial cell layer (**B**), atypical arrangement of the multilayered structure of the epithelium (**C**), and poorly defined Palisades of Vogt (POV; **D**).

Corneal epithelial disorders were detected in both eyes of all patients by slitlamp examination, with their appearance being enhanced by fluorescein staining. Images of two typical staining patterns revealing sheet-like or superficial punctate keratopathy (SPK)–like lesions are shown in Figure 2. A hazy epithelial lesion (sheet-like lesion) extending into the central region of the cornea from the upper and lower limbus was apparent in eight of the 24 eyes, whereas a diffuse, rugged, surface lesion positive for fluorescein staining (SPK-like lesion) was detected in the remaining 16 eyes. Palisades of Vogt (POV), a limbal feature at the border with the conjunctiva that is associated with the presence of corneal epithelial stem cells, are readily detected in normal eyes by slitlamp microscopy [15]. However, they were completely absent in 15 of the 24 eyes and were defective in the remaining nine eyes of the study patients.

### In vivo laser confocal microscopy

**Figure 3:**
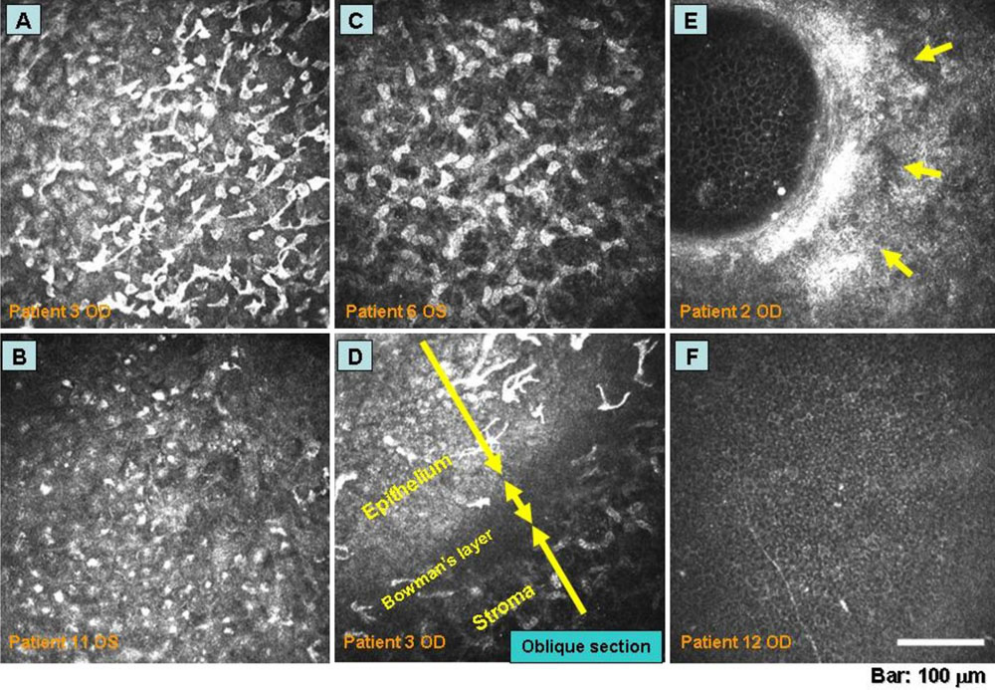
Inflammatory changes in ocular mucositis revealed by in vivo confocal microscopy. In vivo confocal microscopy revealed a high density of dendritic and spherical cells, likely including Langerhans cells, macrophages, monocytes, and neutrophils, in the epithelial basal cell layer (**A**, **B**, **D**) as well as many activated keratocytes in the anterior stroma (**C**) and a reduced number of subepithelial nerve fibers (**F**). Fibrosis (arrows) with a circular pattern of degradation of Bowman’s layer was observed in a case that subsequently showed only minor improvement after discontinuation of S-1 administration (**E**).

Abnormalities of the cornea detected in the study patients by in vivo laser confocal microscopy included: (1) cellular defects or turbulence of superficial epithelial cells; (2) disruption of the layered structure of the epithelium; (3) infiltration of inflammatory cells in the basal cell layer of the epithelium or in the shallow stroma; (4) absence or a reduced number of subepithelial nerves; (5) hyper-reflective granular structures at the center of the epithelial basal cell layer; and (6) changes in the structure of palisades at the limbus (Figure 2, Figure 3, and Table 2). None of these findings were apparent in normal eyes.

**Table 2:** Summary of in vivo confocal microscopic findings in patients with S-1–induced ocular mucositis.

**Findings (20 eyes)**	**Presence (eyes)**	**Absence (eyes)**	**Frequency (%)**
Cellular defect or turbulence of superficial epithelial cells	20	0	100
Disruption of layered structure of the epithelium	20	0	100
Infiltration of inflammatory cells in the epithelial basal cell layer or the shallow stroma	Severe: 4 Moderate: 13	3	85
Partial or complete loss of subepithelial nerves	Complete: 14 Partial: 3	3	85
Hyperreflective granular structures at the center of the epithelial basal cell layer	12	8	60
Changes in the structure of palisades at the limbus	Disappearance : 7 Disruption: 13	0	100

All 20 eyes of the ten study patients examined exhibited corneal epithelial disorders, including cellular defects or turbulence of superficial epithelial cells as well as disruption of the layered structure of the epithelium. Such disorders were thus apparent for both the sheet-like and SPK-like lesions revealed by slitlamp examination. A high density of dendritic or spherical infiltrated cells, likely including Langerhans cells, macrophages, monocytes, and neutrophils, was also observed in the epithelial basal cell layer or the shallow stroma in 17 of the 20 eyes examined. Many activated keratocytes, characterized by a spherical nucleus and reflective cytoplasm, were detected in the anterior stroma of the 17 eyes with a partial or complete loss of subepithelial nerve fibers. The posterior stroma and endothelium appeared normal in all 20 eyes. Hyper-reflective granular structures corresponding to melanocytes are usually observed only in the basal cell layer of the epithelium at the limbus in the normal cornea. Such structures were detected in the central region of the cornea in 12 eyes of the study patients; patients 3, 7, and 9, all of whom did not show an increase in skin pigmentation, did not exhibit infiltration of melanocytes at the central cornea. Palisade structures in the limbal area, likely corresponding to POV, were detected in 13 eyes, even though slitlamp examination failed to detect POV in most of these eyes. However, these structures were defective in all 13 of these eyes and were not detected in the remaining seven eyes examined.

### Histopathologic analysis

**Figure 4:**
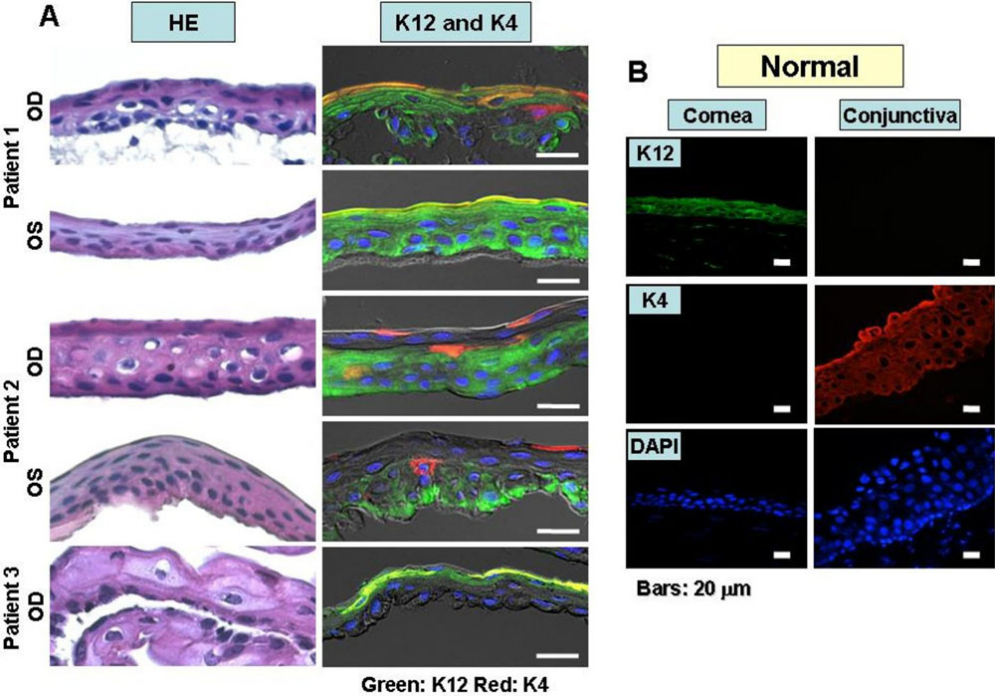
Histopathologic examination of S-1-induced corneal disorders. **A**: Sections were subjected to hematoxylin and eosin (HE) staining and to immunofluorescence staining for both cytokeratin 12 (K12, in green) and cytokeratin 4 (K4, in red). HE staining revealed that each epithelial sheet lacked the stratified structure of the normal corneal epithelium. Immunofluorescence analysis revealed the presence of four types of cells in each lesion: those positive for one, both, or neither of the two cytokeratins. Nuclei were stained with 4′,6-diamidino-2-phenylindole (blue). **B**: K12 was only expressed in corneal epithelium, whereas K4, by contrast, was only expressed in conjunctival epithelium in immunofluorescence staining of the normal human cornea and conjunctiva for K12 and K4.

Frozen sections of the lesions of five eyes of three study patients were examined by HE staining and immunofluorescence staining for cytokeratins (Figure 4). HE staining revealed that each abnormal epithelial sheet lacked the well-organized stratified structure of the normal corneal epithelium (Figure 4A). The cells of each lesion contained nuclei that were irregular in size and shape as well as a vacuolated cytoplasm. The localization of the atypical cells revealed by HE staining appeared to correspond to the localization of the abnormal cells observed with the HRTII-RCM system. Cytological diagnosis of the five specimens by a pathologist yielded one case of moderate dysplasia and four cases of mild dysplasia.

Immunofluorescence staining for both K12 and K4 revealed the presence of four types of cells in each lesion: those positive for one, both, or neither of these cytokeratins (Figure 4A). The specimen from the right eye of patient 1, which was classified as moderate dysplasia, exhibited double-positive (K12 and K4) cells in the superficial cell layer, an isolated K4-positive cell in the wing cell layer, and double-negative cells in the wing and basal cell layers. In the left eye of patient 2, epithelial cells expressing K4 were present sporadically in the superficial and wing cell layers. Cells lacking both K12 and K4 were observed in all layers of the epithelium in both eyes of patient 2 and the right eye of patient 3.

### Progress of the cases

In 11 of the 12 patients, administration of S-1 was terminated with or without instigation of therapy with other antineoplastic drugs. The corneal epithelial disorders of both eyes in nine of these 11 patients healed completely after discontinuation of S-1 treatment, whereas those of both eyes of the remaining two patients were only slightly ameliorated. For the seven of these 11 patients who experienced tearing, canalicular or nasolacrimal duct obstruction was ameliorated in four patients but not in the remaining three patients after discontinuation of S-1 administration.

## Discussion

As far as we are aware, our study provides the first demonstration of S-1-induced ocular mucositis. Our results suggest that this corneal epithelial disorder is related to dysplasia in S-1-treated patients. We noninvasively characterized the pathological features of the lesions with the use of in vivo laser confocal microscopy.

The pathobiology of mucositis had been attributed to direct epithelial injury inflicted over a long period of time. The recent development of an animal model of mucositis [5], however, has indicated that the pathobiology is more complex and involves all components of the mucosa, including connective tissue as well as the epithelium [16]. At the molecular level both pro-inflammatory cytokines and transcription factors are now implicated in the development of mucositis [5]. However, the precise mechanism underlying the development of 5-FU-induced mucositis, especially in humans, remains to be fully determined.

Ophthalmic adverse effects of intravenous 5-FU therapy, such as tearing, eye discharge, and canalicular stenosis, have been reported [6,17,18]. However, S-1-induced corneal disorders appear to be more severe than those associated with other oral 5-FU prodrugs or intravenous 5-FU administration [19]. Given that high concentrations of 5-FU can be achieved for a longer time by administration of S-1, it is likely that adverse effects at the ocular surface are induced by a high concentration of 5-FU in tear fluid or in tissue that results from diffusion of S-1-derived 5-FU from the limbal network of vessels. Further investigation of the relation between the severity of corneal disorders and the concentration of 5-FU in tear fluid is thus warranted.

Among the pathological characteristics of S-1-induced ocular mucositis revealed by in vivo confocal microscopy in the present study, the irregularity of superficial corneal epithelial cell alignment is suggestive of the active migration of epithelial cells to cover epithelial defects, whereas the abnormality in the multilayered structure of the corneal epithelium likely also reflects such defects or incomplete epithelial cell proliferation and differentiation. The toxicity of 5-FU in mouse intestine has been ascribed to both the induction of apoptosis and inhibition of cell proliferation [20], with these effects leading to reduced cellularity in both intestinal crypts and villi. POV at the limbus have a histological structure similar to that of intestinal crypts. S-1 administration was found to result in a reduction in cellularity of POV, as observed by both slitlamp and in vivo confocal microscopy in the present study.

The complete or partial loss of subepithelial nerves observed in the S-1-treated patients in the present study is indicative of nerve destruction or damage associated with an epithelial disorder and inflammation [14,21]. The infiltration of inflammatory cells in the epithelium and the presence of activated keratocytes, which are characterized by reflective nuclei and visible cytoplasmic processes [22], in the shallow stroma of the patients are also indicative of an inflammatory response induced by 5-FU. Cyclooxygenase-2 is upregulated and initiates an inflammatory cascade resulting in activation of matrix metalloproteinases and further tissue damage in cartilage, one of the avascular tissues, such as the cornea [23]. Persistence of inflammation would be expected to result in the development of sight-threatening, irreversible, corneal opacity given that activated keratocytes produce matrix metalloproteinases and contribute to haze induction [22,24].

Corneal epithelial dysplasia is a rare condition that likely arises at the limbus and spreads in a relatively flat manner over the cornea rather than remaining restricted to the limbus or extending into the conjunctiva. The cells of the lesion are atypical in size and shape and they manifest a slight increase in the nucleus-to-cytoplasm ratio, mitotic figures, and disturbance of maturation and polarity [25-27]. Epithelial cells express specific cytokeratins. All corneal epithelial cells thus express K12, and all conjunctival epithelial cells express K4 [28]. Our in vivo confocal microscopic images of the corneal epithelium showed abnormal cells that appeared to correspond to those detected by HE staining of each excised lesion. Immunofluorescence staining revealed four different phenotypes of cells in the excised corneal epithelium of S-1-treated individuals: those positive for one, both, or neither of K12 and K4. The double-positive cells likely represent conjunctival epithelial cells that invaded the cornea via the limbus and adopted characteristics of corneal epithelial cells (corneal epithelial metaplasia). The cells lacking both K12 and K4 likely reflect either undifferentiated cells or epithelial cells that had not achieved tissue-specific cytokeratin expression. The limbus is thought to contain corneal epithelial stem cells [15]. The corneal epithelial disorders persisted in two patients of the present study after discontinuation of S-1 administration, suggesting that corneal epithelial stem cells may also have been affected by S-1 administration in these individuals. Anticancer drugs have previously been shown to alter the expression of cell type-specific cytokeratins [29], and inflammation may affect the expression of junctional proteins in corneal epithelial cells [30]. S-1 thus likely affects the function of corneal epithelial cells, possibly altering their differentiation.

The drug 5-FU and its oral prodrugs, such as S-1, induce the development of pigmented skin lesions [31,32]. We also observed atypical proliferation of melanocytes in the basal cell layer of the corneal epithelium in patients who manifested such skin lesions. The migration of these cells from their normal location at the limbus to the central cornea may be promoted by a deficiency of corneal epithelial cells resulting from the antiproliferative effect of S-1.

Some patients in the present study developed irreversible changes of the corneal epithelium and canalicular or nasolacrimal duct in spite of discontinuation of S-1 administration. These findings suggest that it is important to detect the adverse effects of S-1 early in order to prevent the development of such irreversible changes and progression of ocular surface disorders. Good vision depends on corneal transparency and smoothness, and it is important for maintenance of quality of life in individuals with tumors.

In conclusion, we have described for the first time the development of ocular mucositis in patients treated with S-1. In vivo confocal microscopy revealed that the corneal disorders of S-1-treated patients were characterized by disruption of the normal layered structure of the corneal epithelium in association with inflammation. In vivo confocal microscopy may thus prove useful as a noninvasive means to elucidate the pathogenesis of adverse effects of S-1. Its application to the early detection of adverse effects of S-1 at the ocular surface might also inform intervention to prevent progression of such effects not only at the ocular surface but also in other tissues.
